# Case report: Squamous cell carcinoma of the prostate-a clinicopathological and genomic sequencing-based investigation

**DOI:** 10.3389/pore.2023.1611343

**Published:** 2023-11-28

**Authors:** Caixin Zhang, Yong Jia, Qingnuan Kong

**Affiliations:** ^1^ Department of Pathology, Qingdao Municipal Hospital, Qingdao, China; ^2^ Department of Urology, Qingdao Municipal Hospital, Qingdao, China

**Keywords:** squamous cell carcinoma, prostate adenocarcinoma, whole exome sequencing, gene mutation, SCC

## Abstract

Squamous differentiation of prostate cancer, which accounts for less than 1% of all cases, is typically associated with androgen deprivation treatment (ADT) or radiotherapy. This entity is aggressive and exhibits poor prognosis due to limited response to traditional treatment. However, the underlying molecular mechanisms and etiology are not fully understood. Previous findings suggest that squamous cell differentiation may potentially arise from prostate adenocarcinoma (AC), but further validation is required to confirm this hypothesis. This paper presents a case of advanced prostate cancer with a combined histologic pattern, including keratinizing SCC and AC. The study utilized whole-exome sequencing (WES) data to analyze both subtypes and identified a significant overlap in driver gene mutations between them. This suggests that the two components shared a common origin of clones. These findings emphasize the importance of personalized clinical management for prostate SCC, and specific molecular findings can help optimize treatment strategies.

## Introduction

Squamous differentiation of prostate cancer is rare, accounting for less than 1% of all prostate cancer cases [1]. It arises after conventional androgen deprivation treatment (ADT) or radiotherapy, though there have been occasionally reported cases of *de novo* squamous cell carcinoma (SCC) [2]. Clinically, SCC of the prostate exhibits aggressive behavior and is associated with poor prognosis due to its limited response to standard treatment for prostate adenocarcinoma [2]. Furthermore, the etiology and underlying molecular mechanisms remain incompletely understood.

Genome profiling is a valuable tool for origin identification, etiology investigation, and targeted therapy selection. By now, only four studies have documented molecular alterations in SCC of the prostate. In all of these studies, activation of the ERG pathway has been investigated, with three cases presenting a *TMPRSS2*-*ERG* fusion and one case showing a *SPOP* mutation (Table 1). These findings suggest that squamous cell differentiation may potentially arise from prostate adenocarcinoma (AC) [1–3]. Nevertheless, further validation is required to confirm this hypothesis.

**TABLE 1:** Summary of genetic alterations identified in the previous cases.

	Genetic alterations	References
Case #1	*SPOP* F133V	Pal, S. K., et al. (2020) [1]
	*TET2* K113fs*15	
	*PTEN* loss	
Case #2	*TMPRSS2-ERG* fusion	Pal, S. K., et al. (2020) [1]
	*PTEN* T319fs*3	
Case #3	*TMPRSS2-ERG* fusion	Clark, M., et al. (2020) [2]
	*PTEN* homozygous loss	
	*RB1* homozygous loss	
	*CARD11*: c.383C > T (p.T128M)	
	*CTNNB1*: c.98C > G (p.S33C)	
Case #4	*TMPRSS2-ERG* fusion	Ali, S. M., et al. (2017) [3]
	*PTEN* homozygous loss	
	*TP53* V147G	
	*CDKN2A* loss	
	*CDKN2B* loss	

In this paper, we reported a case of advanced prostate cancer with a combined histologic pattern after ADT, including keratinizing SCC and AC. The study utilized whole-exome sequencing (WES) data to analyze both subtypes and identified a significant genetic overlap between them. These findings support previous research suggesting that squamous differentiation may originate from the same origin of clones as prostate adenocarcinoma. However, additional investigation is required to comprehensively comprehend the molecular pathways implicated in squamous transformation.

## Case report

**FIGURE 1:**
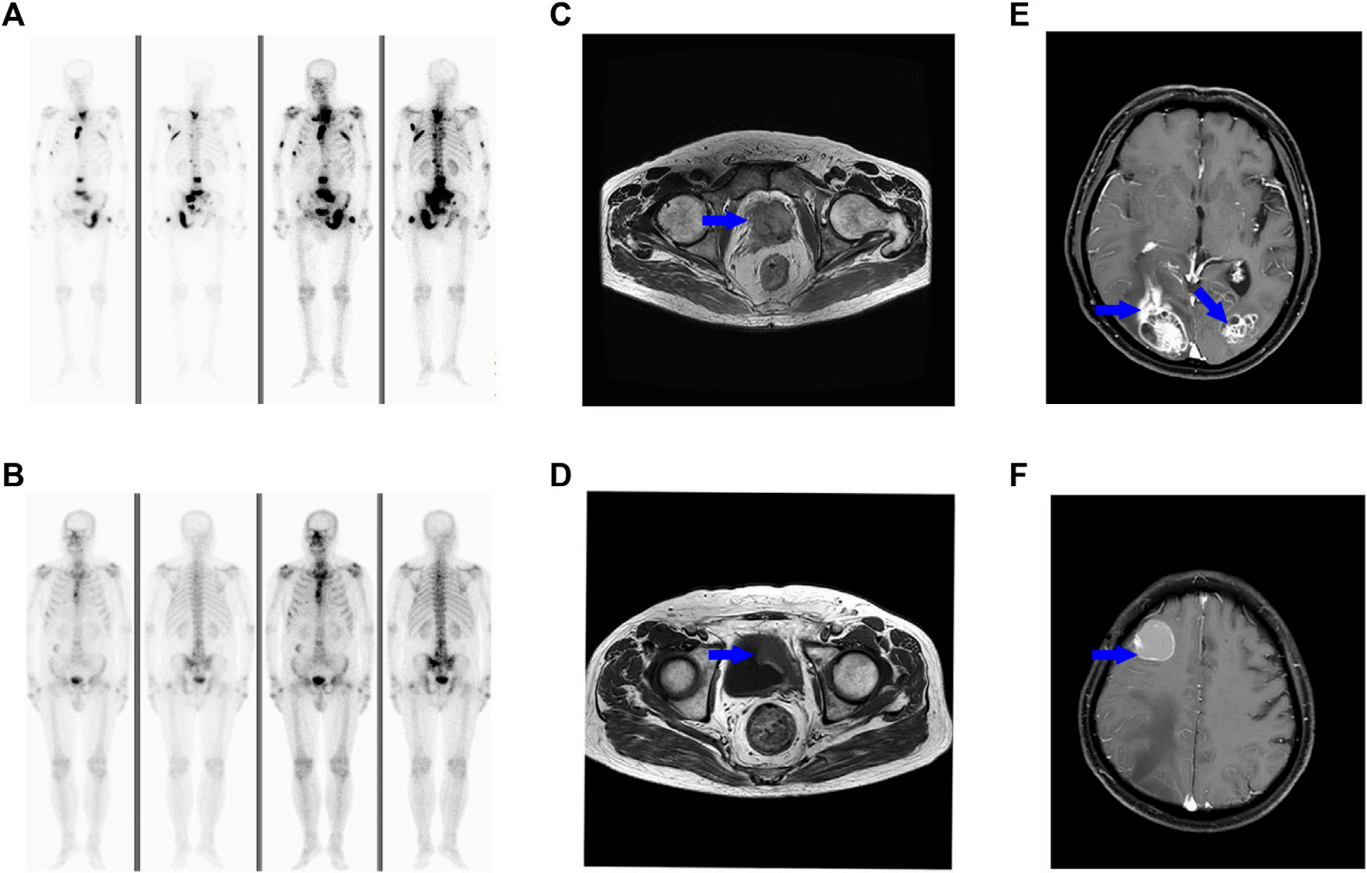
Bone scan shows the patient developed multiple loci of metastasis in the lumbar vertebra, sacral vertebra and left pubis **(A)**. The metastatic lesions response well to ADT **(B)**. MR images show a soft tissue mass in the pelvis [**(C)**, blue arrow], and the lesions partially regress after three cycles of chemotherapy [**(D)**, blue arrow]. A brain MRI scan reveals multiple metastases [**(E, F)**, blue arrow].

**FIGURE 2:**
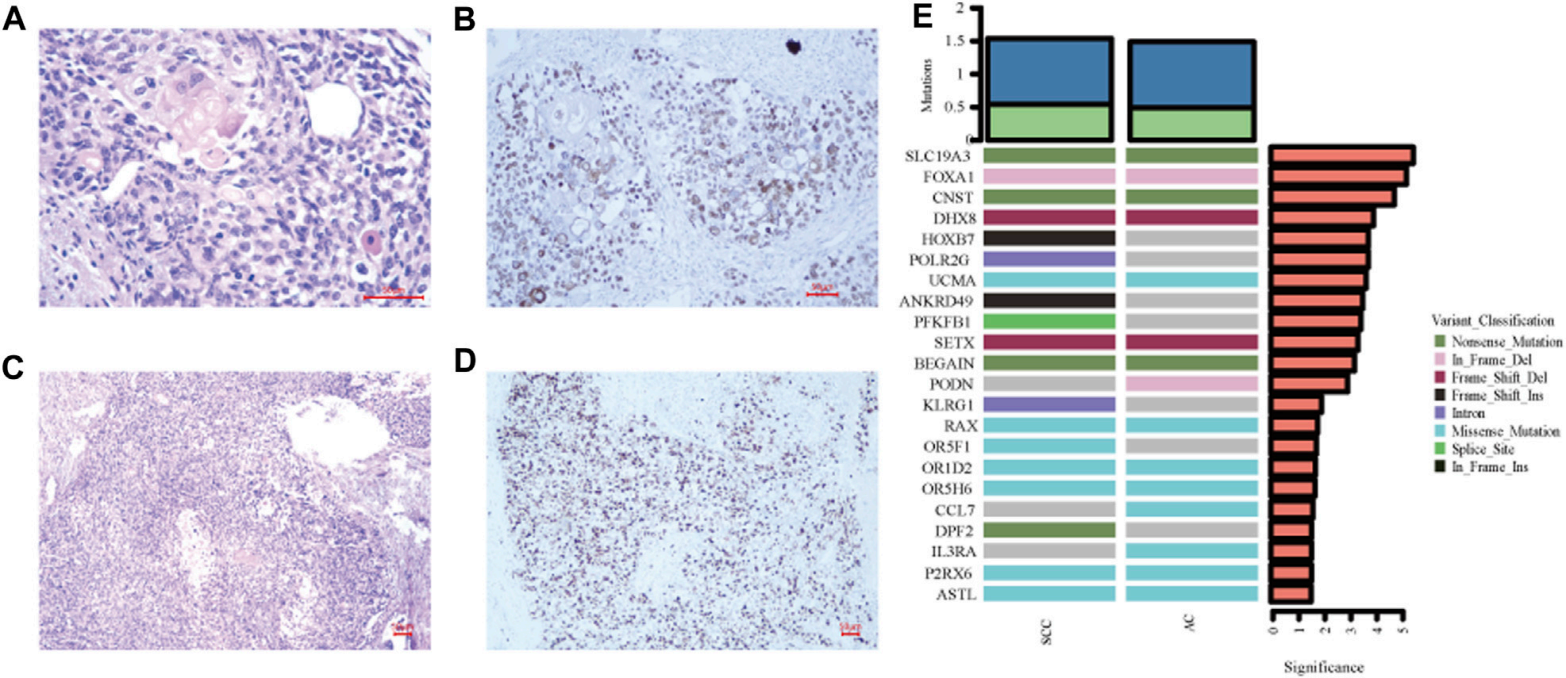
Histological examination and immunohistochemistry results. **(A)** The squamous component is composed of keratinized carcinoma nests with polygonal cells containing voluminous cytoplasm (Hematoxylin and eosin, ×400). **(B)** P40 staining shows nuclear positivity for the squamous carcinoma cells (immunohistochemistry staining, ×200). **(C)** The prostate adenocarcinoma displays a Gleason score of 5 with a solid pattern (Hematoxylin and eosin, ×100). **(D)** The expression of prostate-specific marker NXK3.1 confirms the diagnosis (immunohistochemistry staining, ×100). **(E)** Comparison of genes with high mutation frequencies between squamous cell carcinoma and adenocarcinoma.

A 69-year-old male with elevated serum prostate-specific antigen (PSA) (57.93 ng/mL) was referred to our hospital in December 2018. The bone scan images revealed multiple lesions in the lumbar vertebra, sacral vertebra and left pubis, consistent with metastases (Figure 1A). Ultrasound-guided random systematic six quadrant needle biopsy was performed. Pathological examination showed a high-grade prostate adenocarcinoma involving 5/12 biopsy cores, including Gleason score of 5 + 5 = 10 in two cores, 4 + 5 = 9 in one core, and 3 + 4 = 7 in two cores. Then he underwent orchiectomy and received ADT with leuprorelin. His serum PSA level remarkably reduced and the follow-up bone scan showed a reduction in metastatic lesions in May 2019 (Figure 1B). However, the PSA level increased to 1.83 ng/mL in November 2019, consistent with biochemical recurrence, and abiraterone was administered at that time. For several months thereafter, the disease remained stable with PSA level ranging from 0.116 to 0.190 ng/mL until he presented intermittent painless hematuria in December 2020. The repeated needle biopsy revealed prostate adenocarcinoma with a Gleason score of 5 + 5 = 10 in three cores, as well as a Gleason score of 5 + 4 = 9 in one core. Additionally, to alleviate the urinary tract obstruction, the patient underwent a trans-urethral resection of the prostate (TURP). Pathological examination on TURP samples displayed two distinct histologic patterns: SCC and AC. The keratinizing SCC component was characterized by irregularly infiltrating cancer nests that exhibited notable keratin pearls at low magnification and pleomorphic polygonal cells containing abundant eosinophilic cytoplasm at high magnification (Figure 2A). The AC morphology demonstrated a high-grade prostate cancer, which was assigned a Gleason score of 5 + 4 = 9 (Figure 2C). The immunohistochemical assays were carried out on a BenchMark ULTRA automated immunohistochemical slide staining system (Ventana Medical Systems, Inc.) using the following primary antibodies: PSA (clone MX030, 1:80, Maixin Biotech), NKX3.1 (clone EP356, Maixin Biotech), P40 (clone ZR8, Maixin Biotech), MLH1 (clone M1, ready to use, Ventana), PMS2 (clone MX061, 1:100, Maixin Biotech), MSH2 (clone A16-4, ready to use, Ventana), MSH6 (clone SP93, ready to use, Ventana) with positive and negative controls. The SCC was negative for PSA and NKX3.1, but positive for P40 (Figure 2B). In contrast, the adenocarcinoma component demonstrated diffuse positivity for prostate-specific marker NKX3.1 (Figure 2D). Notably, both components showed retained staining for the four mismatch repair proteins (MMR) (MLH1, MSH2, MSH6, and PMS2), referring to a status of MSI-L.

Sections representing the SCC and AC components were sent separately for WES analysis (Data are available on Mendeley Data, V1, doi: 10.17632/zfv39hddv2.1). Briefly, genomic DNA was extracted using QIAGEN GeneRead DNA FFPE Kit, according to the manufacturer’s instructions. The DNA quality was confirmed before shearing it with a Covaris sonicator (LE220) (Covaris, Woburn, MA, United States). The gDNA library was established by a Roche NimbleGen SeqCap EZ Exome V3/Agilent SureSelect Human All Exon V6 kit, and sequenced on an Illumina PE150. The reads were aligned to the GRCh37 build of the human reference genome.^1^ The average coverage depth of WES across the targeted regions was 127x in AC and 169x in SCC. The tumor mutation burden (TMB) was similar between the two subtypes with 1,327 mutations (including 881 SNVs and 447 Indels) identified in AC and 1,224 mutations (including 831 SNVs and 393 Indels) in SCC. Additionally, all somatic mutations were matched to known driver genes reported in the Cancer Gene Census as well as three relevant articles [4–6]. The results showed there was a complete genetic overlap in driver gene mutations between SCC and AC, including SNVs in *BRCA1* and *BLM*, as well as Indels in *FOXA1* and *SETX* (Table 2). The study also examined genes that had a high frequency of mutation. The SCC component showed differential mutant genes including *HOXB7*, *POLR2G*, *ANKRD49*, *PFKFB1*, *KLRG1*, *OR5F1*, and *DPF2*, when compared to the AC (Figure 2E).

**TABLE 2:** Details of mutant genes.

Genes	Chr	Mutations	Type	Variant allele frequencies (%)	CGC cancer types
BRCA1	17	NM_007297:exon9:c.G3702C:p.Q1234H	Non-synonymous SNV	16.1	Ovarian (somatic)
BLM	15	NM_000057:exon16:c.A3167G:p.K1056R	Non-synonymous SNV	39.5	Leukemia, lymphoma, skin squamous carcinoma, other tumor types (germline)
FOXA1	14	NM_004496:exon2:c.754_774del:p.252_258del	Non-frameshift deletion	17.3	breast, prostate (somatic)
SETX	9	NM_001351527:exon10:c.4301_4310del:p.D1434fs	Frameshift deletion	26.7	Undertermined

The patient refused to receive Olaparib treatment and was lost to follow-up for about 1 year and a half. When he revisited our hospital in July 2022, the CT scan revealed a large soft tissue mass in the pelvis (Figure 1C). There were no changes observed on the bone lesions. Then the patient received eight cycles of systematic chemotherapy with docetaxel/carboplatin. The pelvic mass responded well to treatment and partially regressed by the end of the third cycle in October 2022 (Figure 1D). But elevated serum PSA was observed from the fifth cycle. Additionally, the patient began to display mild cognitive impairments and abnormal consciousness. In April 2023, multiple metastatic foci were discovered in the brain, confirming tumor progression (Figures 1E, F).

## Discussion

When prostate cancer develops a progressive phenotype after failure of anti-hormonal therapy, the emergence of SCC is extremely rare [7]. Unfortunately, conventional treatments show limited success in treating this distinct entity, leading to an unfavorable outcome [8]. Therefore, further studies are needed to identify the underlying molecular alterations for improving clinical treatment approaches. We reported a patient with progressed prostate cancer that exhibited a combination of SCC and AC histological patterns. Analysis of their WES data revealed that both samples shared the same mutations in *FOXA1*, *BRCA1*, *BLM*, and *SETX*. The results indicated that multiple molecular mechanisms, such as activation of the AR pathway, genomic instability, and abnormal accumulation of R-loops, may contribute to the development of the two subtypes. Moreover, the overlapping molecular alterations suggest a common origin of clones from which they arise, consistent with previous findings which indicated that there are genetic similarities identified in both SCC and primary prostate AC.

*FOXA1* is the third most frequently mutated genes in the western cohorts with a frequency of 11%, but ranks first in the Chinese cohorts with a frequency of 41% [9]. As a pioneer factor, it is recruited to the chromatin to facilitate the sequential binding of other transcriptional factors including AR [10]. Mutations in *FOXA1* can lead to dysregulation of the AR pathway and have been implicated in both the initiation and progression of prostate cancer. According to a study by [11], there are three classes of *FOXA1* mutations. Class 1 mutations occur within the Wing2-region (247aa–269aa) of the DNA-binding Forkhead domain (FKHD) and act as AR activation mutations by enhancing chromatin mobility and binding frequency. Meanwhile, both class 2 mutations and class 3 rearrangements involve transcripts promoters. Class 2 mutations consist of C-terminal domain truncations, while class 3 rearrangements include structural variants such as duplications and translocations. The *FOXA1* (NM_004496:exon2:c.754_774del:p.252_258del) mutation observed in this case belongs to the first class of mutations, which suggests the involvement of AR pathway activation. In addition, *FOXA1* mutants have been shown to alter normal luminal epithelial differentiation in prostate cells. Adams et al examined fourteen *FOXA1* mutants mapping to the forkhead (FKHD) DNA-binding domain. Of which, there are two R219 mutants identified to activate neuroendocrine transcriptional program, while the others promoted luminal differentiation [9]. However, it is still unclear whether the *FOXA1* mutation identified in this case can induce squamous differentiation, and further investigation is required.

Furthermore, it is believed that somatic mutations of *BRCA1* and *BLM* hinder DNA damage repair and result in genomic instability. The loss of somatic *BRCA1* is linked to advanced tumor stage and lower disease-free survival rates in patients undergoing radical prostatectomy [12]. Germline *BRCA1* mutations are present in 0.87%–1.25% of prostate cancer while somatic mutations account for less than 1% [13]. Based the NCCN guideline for prostate cancer (Version 1.2023) released in 2022, individuals with germline or somatic *BRCA1* gene mutations may benefit from olaparib treatment, particularly if they have previously undergone treatment with enzalutamide or abiraterone.

*BLM* is a RecQ DNA helicase that acts as a key player in maintaining the DNA damage repair response. It is widely recognized that biallelic loss-of-function BLM mutations lead to Bloom syndrome [14]. *BLM* is found to overexpress in prostate cancer and has been shown to promote proliferation, migration, and invasion of PC3 cells [15]. However, whether somatic *BLM* mutations contribute to the risk of prostate cancer is unclear. After analyzing the genetic data of 796 patients, Elisa M. Ledet et al. did not find any associations between somatic mutations of *BLM* and metastatic prostate cancer [16]. Nevertheless, it is well known that *BLM* is a major subunit of BASC (BRCA1-associated genomic surveillance complex). *BLM* and *BRCA1* work together in regulating post-replicational DNA repair processes by colocalizing with PCNA-positive replication forks in mid to late S phase cells [17]. In consequence, defects in both *BLM* and *BRCA1* genes lead to dysfunction of the BASC, ultimately causing genomic instability.

In addition to AR pathway deficiency and genomic instability caused by BASC disruption, the presence of the *SETX* mutation suggests that R-loop mediated genome instability may be involved. R-loop is a unique nucleic acid structure composed of a DNA:RNA hybrid and a single strand of DNA [18]. It has crucial roles in modulating gene transcription, DNA and histone modifications, DNA replication, and genome stability. The formation of R-loop is a dynamic process mediated by several RNA binding and processing factors. Among these factors, *SETX*, a known DNA:RNA helicase, resolves R-loops through directly unwinding DNA:RNA hybrids, then degrades the released RNA [18]. Dysfunctional *SETX* causes abnormal accumulation of R-loops due to impaired degradation, making DNA more sensitive to damaging agents and contributing to genomic instability.

*SETX* has been identified as a binding partner for *BRCA1* and can participate in the termination region of R-loop transcription [19]. When the *BRCA1/SETX* complex is disrupted, unrepaired ssDNA is broken, resulting in transcriptional-associated genomic instability [20]. *SETX* mutations are found in approximately 4% of metastatic prostate cancer and 1.2% of prostate adenocarcinoma [21]. Although the relationship between *SETX* and prostate cancer has not been well elucidated, recent research showed that depletion of *SETX* leads to an increase in the accumulation of cytoplasmic RNA-DNA hybrid fragments, which can activate the innate immune response and contribute to various diseases, including cancer [22]. Mutations in *SETX* are linked to improved survival rates in patients with lung adenocarcinoma [23]. Further investigation is needed to fully understand the significance of genome instability caused by dysfunctional *SETX* as well as abnormal R-loop accumulation in the evolution of advanced prostate cancer.

There have been several hypotheses proposed to explain the emergence of squamous transformation in the prostate. One theory suggests that squamous differentiation may arise from squamous cell metaplasia of the urothelial epithelium, which can be triggered by inflammation or manual instrumental manipulation [24]. Another possible origin for squamous differentiation is pluripotent stem cells in the prostate ducts, which can transform into squamous cells under certain circumstances [25]. The overlapping driver gene mutations investigated in the present case suggest that SCC and AC may have originated from the same progenitor cells, supporting previous assumptions. However, specific pathways that govern the differentiation of squamous cells are not fully understood. Seven differential genes with high mutation frequency have been identified in SCC in the present case. Among them, *HOBX7* was reported to be associated with tumor aggressiveness and unfavorable prognosis in head and neck squamous cell carcinoma (HNSCC). Knockdown of *HOXB7* hindered tumor cell proliferation, migration and invasion by inducing cell apoptosis in HNSCC cell lines [26]. These findings highlighted the functions of *HOBX7* as a crucial regulator in tumor growth, but its involvement in SCC differentiation remains uncertain.

## Conclusion

This paper presents a case study of advanced prostate cancer with a combined histologic pattern after ADT, including keratinizing SCC and AC. The study identified a significant genetic overlap between the two, supporting previous research that suggests SCC may originate from the same clones as prostate adenocarcinoma. Moreover, molecular pathways involved in the squamous transformation are required for further investigation. The findings emphasize the necessity for individualized clinical management of prostate SCC and suggest that specific molecular findings could help optimize treatment strategies.

## Data Availability

The datasets presented in this study can be found in online repositories. The names of the repository/repositories and accession number(s) can be found in the article/Supplementary Material.

## Footnotes

1
http://grch37.ensembl.org/Homo_sapiens/Info/Index
